# Stress as a Chromatin Landscape Architect

**DOI:** 10.3389/fcell.2021.790138

**Published:** 2021-12-14

**Authors:** Anastassiia Vertii

**Affiliations:** Department of Molecular, Cellular and Cancer Biology, University of Massachusetts Medical School, Worcester, MA, United States

**Keywords:** stress, 3D chromatin, inflammation, topologically associated domains, chromatin loops, heat shock, mechanical stress, chromatin territories

## Abstract

The exponential development of methods investigating different levels of spatial genome organization leads to the appreciation of the chromatin landscape's contribution to gene regulation and cell fate. Multiple levels of 3D chromatin organization include chromatin loops and topologically associated domains, followed by euchromatin and heterochromatin compartments, chromatin domains associated with nuclear bodies, and culminate with the chromosome territories. 3D chromatin architecture is exposed to multiple factors such as cell division and stress, including but not limited to mechanical, inflammatory, and environmental challenges. How exactly the stress exposure shapes the chromatin landscape is a new and intriguing area of research. In this mini-review, the developments that motivate the exploration of this field are discussed.

## Introduction

The spatial organization of chromatin in interphase cells aims for the correct expression of cell type-specific genes and their accessibility to the regulatory elements. Multiple levels of 3D genome organization achieve this specificity. The initial level of 3D chromatin organization, chromatin loops, are interactions between enhancers and promoters marked by CCCTC-binding factor (CTCF)–binding sites, which are laid inside topologically associated domains (TADs) (Kempfer and Pombo, 2020). At the next level, interactions between TADs forming transcriptionally active euchromatin (A) and silenced heterochromatin (B) compartments have been detected in the Hi-C experiments (Kempfer and Pombo, 2020). Heterochromatin drives the spatial organization of the interphase nucleus (Falk et al., 2019) and includes lamina-associated domains (van Steensel and Belmont, 2017) and nucleolus-associated domains, including pericentric heterochromatin, which is also often found at the chromocenters in mouse cells (Guenatri et al., 2004; Németh et al., 2010). The proximity of the chromatin domains to different nuclear substructures often regulates chromatin transcriptional activity—for example, association with nuclear speckles, subnuclear structures enriched in pre-mRNA splicing factors (Lamond and Spector, 2003)—is a characteristic of transcriptionally active chromatin (Kim et al., 2020). By contrast, gene-poor and transcriptionally inactive heterochromatin regions, including pericentric heterochromatin, are often found at the lamina and nucleolar peripheries (Németh et al., 2010; Kind et al., 2015; Quinodoz et al., 2018). At the most global level, chromatin is organized into cell type–specific chromosome territories, a spatial map within the interphase nucleus (Meaburn and Misteli, 2007; Fritz et al., 2019).

Although methods to study the 3D chromatin organization are not the subject of this work and have been reviewed elsewhere (Kempfer and Pombo, 2020), the importance of single-cell methods in studying stress response should be appreciated, as the response may vary depending on the cell cycle stage and other factors. Dividing cells have to re-establish the abovementioned levels of the organization, erased in mitotic chromosomes. Moreover, different cell types, carrying the same genome, demonstrate differential segregation of the genome into A (euchromatin) and B (heterochromatin) compartments, represented by Active- and Inactive Nuclear Compartments at the microscopy level and varying in their proximity to liquid phase separated nuclear bodies. (Cremer et al., 2017). Such “chromatin breathing” provides the basis for differentiation and formation of different tissues in multicellular organisms (Meshorer et al., 2006). Additionally, the exposure to various stress such as inflammation changes the gene expression profile, and cell function and fate. Whether changes are limited to gene expression or include 3D chromatin organization is an advancing field. Below, the examples of stress exposure that alter chromatin architecture are discussed.

## Heat Stress and Chromatin Architecture

Heat stress (HS) is an *in vitro* tool to model febrile conditions or heat stroke. Febrile condition is one of the major signs of inflammation and an evolutionarily conserved feature of the immune response for over 600 million years (Evans et al., 2015). While the terms febrile conditions, pyrexia, fever, and hyperthermia are generally interchangeable, hyperthermia is often referred to as environmental HS such as heat stroke in contrast to the brain-regulated elevation of body temperature due to the effects of external (pathogenic microorganisms) or internal (pro-inflammatory cytokines) pyrogens on the hypothalamus (Ushikubi et al., 1998; Walter et al., 2016; Prajitha et al., 2018). The febrile condition is defined as an increase in core body temperature above 38.3°C. The most profound systemic effects of hyperthermia impair the gastrointestinal tract, heart, kidney, and brain functions (Walter et al., 2016). Although the kinetics of heating between the *in vitro* and pyrogen-induced heat is not compared, HS between 39 and 44°C is physiologically relevant (Glazer, 2005) with 39–42°C being frequent and 42°C reported for initial isolation of mammalian heat shock proteins (Welch and Feramisco, 1982), suggesting 42°C as the optimal in *in vitro* treatment.

The detrimental long-term consequences of hyperthermia are well described (Mégarbane et al., 2003; Iwashyna et al., 2010; Walter and Carraretto, 2016; Barichello et al., 2019); however, the cellular mechanisms that explain these changes are not understood. The exposure to HS activates heat shock factor 1 (HSF1), initiating the HSF1-dependent and HSF1-independent transcriptional alterations (Mahat et al., 2016). Massive transcriptional changes are accompanied by alterations in 3D chromatin architecture at distinct levels of chromatin organization. Specifically, HS activates long-distance movements of HSP70 loci toward the speckles, leading to gene activation (Jolly et al., 1999; Khanna et al., 2014). The movement relies on nuclear actin polymers because de-polymerization of actin prevents the speckle association and the consequent activation of Hsp70 transgene (Khanna et al., 2014). Hsp70 gene is one of the best-studied genes in terms of chromatin remodeling during transcriptional activation, and besides stress, a mood stabilizing and anticonvulsant drug, valproic acid, increases H3K4me2 of Hsp70 promoter and induces Hsp70 transcription in neurons (Marinova et al., 2011). Additionally, a number of studies have detected HS- and HSF1-dependent activation of typically silent constitutive heterochromatin regions, the satellite repeats on human chromosome 9 (Jolly et al., 2002, 2004; Rizzi et al., 2003; Valgardsdottir et al., 2008; Sengupta et al., 2009; Eymery et al., 2010; Pezer and Ugarkovic, 2012; Feliciello et al., 2015; Col et al., 2017; Feliciello et al., 2020). These repeats are often closely associated with the nucleoli periphery, one of the major locations of heterochromatin domains within the nucleus (Politz et al., 2016; Vertii et al., 2019; Bersaglieri et al., 2020; Bury et al., 2020; Wang et al., 2021). Satellite III repeats are located in the pericentromeric regions of acrocentric chromosomes and in response to HS produce long noncoding RNA transcripts that accumulate at the site of transcription, primarily at chromosome 9, and help to mediate HS response. These sites are also called nuclear stress bodies (Jolly et al., 2002; Rizzi, 2003; Jolly et al., 2004). Although dissociation from the nucleoli of a transgene is associated with activation (Fedoriw et al., 2012), whether the activation of satellite repeats requires dissociation from the nucleoli remains to be investigated. Thus, HS alters constitutive heterochromatin organization at satellite repeats and active Hsp70 loci.

Chromatin undergoes HS-dependent changes not only in the human satellite repeats and Hsp70 transgenes but also in *Drosophila* embryos (Seong et al., 2011). In this organism, the epigenetic inheritance of the heterochromatin alterations occurs after HS-induced activation of a mitogen-activated protein kinase (MAPK) p38 and its downstream target transcription factor ATF-2 (Seong et al., 2011). Upon phosphorylation by p38, ATF-2 is released from the H3K9me2-enriched heterochromatin regions and heterochromatin linker protein HP1, decreasing H3K9me2 and disrupting heterochromatin in the early embryogenesis (Seong et al., 2011). Unlike the role of ATF-2 in heterochromatin organization, stress enhances the binding of phospho-ATF-2 to the promoter sites of the target genes (Seong et al., 2011). Notably, the stress activation of p38 is typically a very short-term event (less than 30 min), suggesting that short-term stress has the potential for long-term consequences. Thus, interactions of both euchromatin and heterochromatin regions with nuclear structures are altered by HS. However, the study of human K-562 and *Drosophila* cells using the Hi-C method revealed striking stability of A and B chromatin compartments and TADs (Ray et al., 2019), suggesting a level of stability, possibly securing the platform for the stress response. For example, the HSF-1 and its target genes are found within the same TADs, providing a “premade” template for the fast response.

In human embryonic stem cells, HS elicited changes are mediated by the chromatin loop alterations impacting promoter–enhancer interactions (Lyu et al., 2018). These alterations involve the MAPK kinase JNK pathway–activated transcription complex, activator protein (AP-1), and pluripotency factors. Based on the ethynyluridine sequencing (EU-seq) method that assesses nascent transcripts, a massive transcriptional tsunami in response to 60 min of 43°C HS in human H9 embryonic stem cells involving the activation of 2,506 genes and suppression of 1,610 genes occurs (Lyu et al., 2018). These data are paired with 7,576 HS-gained and 11,232 control-lost enhancers, implying structural changes in the chromatin organization. Intriguingly, a significant number of HS-altered enhancers do not contain HSF1 motif but include motifs for AP-1 (for activated enhancers), pluripotency factors such as NANOG and OCT4 (for decommissioned enhancers), and architectural proteins such as DNA-binding protein CTCF (Lyu et al., 2018). CTCF, together with cohesin, mediates long-range chromatin loops and defines TAD borders (Wutz et al., 2017). Temperature stress induces changes in CTCF occupancy and, associated with these changes, alters promoter–enhancer interactions, thus pointing at the TAD border dynamics during stress exposure. Another study elucidated stress-induced 3D chromatin alterations by focusing on changes in facultative heterochromatin modifier, polycomb repressive complexes PRC1 and PRC2 (Azkanaz et al., 2019). Specifically, after the exposure of K562 cells to 60 min of 44°C, the components of the polycomb repressive complexes (PRC) were sequestered into the non-membranous nuclear organelle, the nucleolus. The sequestration of the PRC proteins into the nucleoli happens concomitantly with the decrease in PRC binding to target genes. Moreover, the accumulation, although reversible, of PRC in the nucleolus correlates with the loss of H3K27me3, a hallmark of facultative heterochromatin (Azkanaz et al., 2019). The recovery of PRC proteins from nucleolar localization depends on the molecular chaperones Hsp70 and DNAJB1 activity (Azkanaz et al., 2019). This study suggests the possibility of heterochromatin remodeling and “epigenetic instability” during the HS exposure and recovery window. Moreover, the nucleoli act as a stress sensor changing its protein composition and function in response to stress (Boulon et al., 2010; Frottin et al., 2019). Close to 200 proteins have been sequestered into the nucleolus upon HS to prevent irreversible aggregation, and the recovery requires Hsp70 chaperone machinery (Frottin et al., 2019). This study supports the notion that proteins translocate into the nucleoli upon stress and require Hsp70 for recovery and suggests massive but dynamic restructuring of the nucleolar proteome during stress and possible sensitivity of the surrounding chromatin domains as a consequence.

**FIGURE 1:**
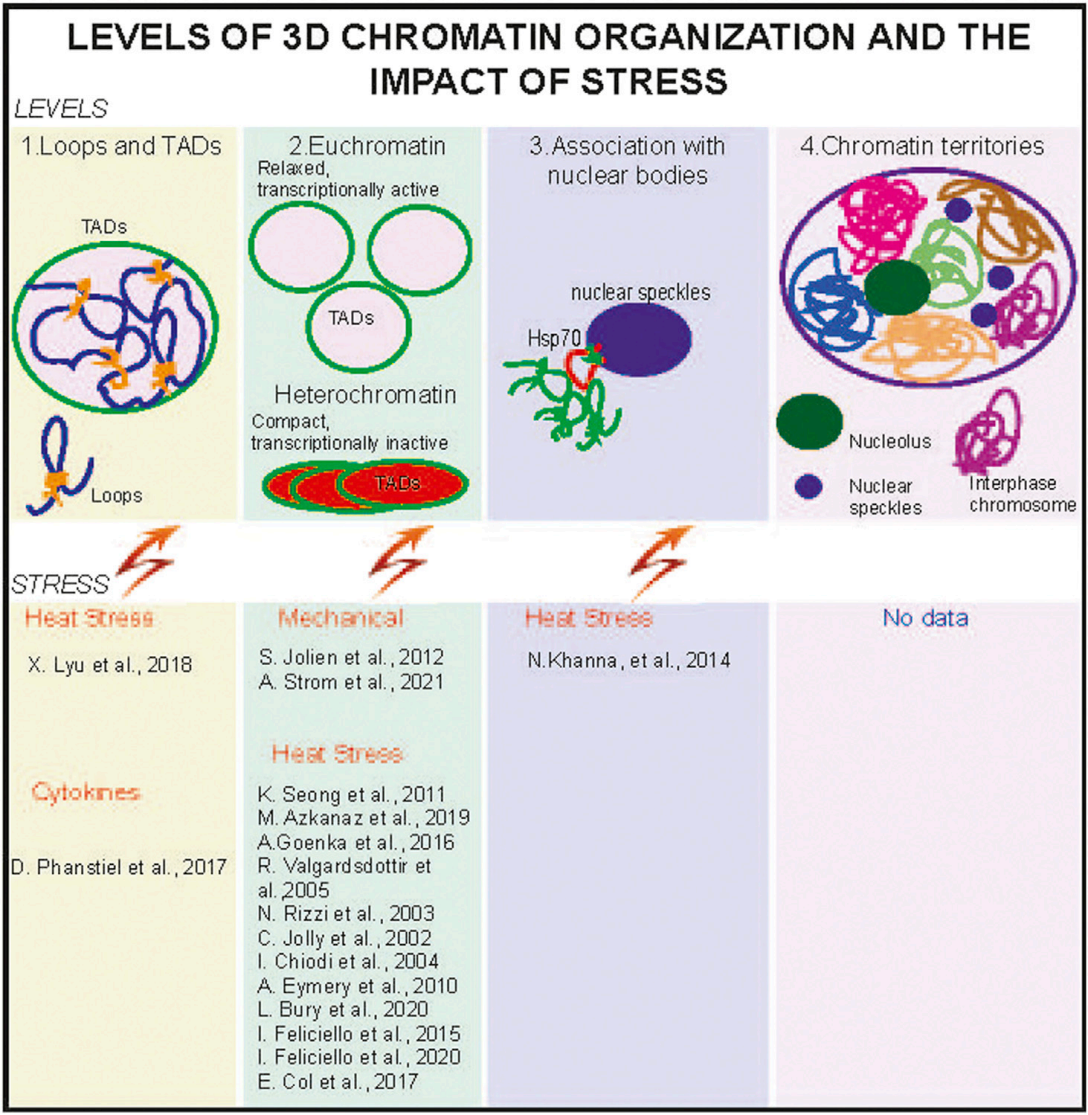
Stress alters 3D chromatin organization at different levels. 3D chromatin is organized at the levels of chromatin loops and topologically associated domains. TADs (1), relaxed and transcriptionally active euchromatin (compartment A), and compact and transcriptionally silenced heterochromatin (compartment B) (2). Euchromatin regions are often found in the nuclear interior and proximal to nuclear speckles (3). Heterochromatin preferentially localizes at the nuclear and nucleolus periphery. Individual chromosomes form chromosome territories. CT, in interphase nuclei (4), and no data about the stress effects on CTs are available. TADs, heterochromatin, and euchromatin were reported to be affected by several stresses **(lower panels)**.

In summary, HS alters 3D chromatin organization at the level of promoter–enhancer interactions and TAD borders, nuclear speckles, and heterochromatin organization (Figure 1). These changes depend on the cell type and methods used, sparking some controversy and questions about the limitations of the methods.

## Inflammatory Cytokines Change 3D Chromatin Organization in Endothelial and Immune Cells

Inflammation is a commonly experienced stress for most organisms. Immune cells, in response to inflammatory stimuli such as bacterial lipopolysaccharides or others, secrete pro-inflammatory cytokines into nearby tissues and the blood. The pro-inflammatory cytokines not only induce febrile condition but also activate stress kinase pathways in the immune and nonimmune cells, leading to amplification of response. Chronic conditions such as diabetes result in the presence of pro-inflammatory cytokines in the bloodstream, constantly affecting juxtaposed cells. The endothelial cells line the blood vessels and are one of the first to be exposed to stress. A recent study looked at the 3D chromatin alterations in the endothelial cells in the presence of a combination of components that mimic inflammatory response causing endothelial dysfunction during diabetes mellitus, namely, high glucose and pro-inflammatory cytokine tumor necrosis factor alpha (TNF-alpha) (Calandrelli et al., 2020). The induced endothelial dysfunction was assessed by single-cell RNA-sequencing and further analyzed by the Hi-C method for DNA–DNA interactions, while RNA–DNA interactions were evaluated by the iMARGI method (Calandrelli et al., 2020). RNA–DNA interactions were further confirmed by using samples from diabetic patients. Notably, the Hi-C method did not reveal significant differences in DNA–DNA interaction between stressed and control endothelial cells, but the main finding of the changes in chromatin-associated RNA suggests its pivotal role in DNA organization in dysfunctional endothelium. The exposure of the endothelial cells to TNF-alpha activates micro RNA mir-3679-5p, which in turn leads to lysine demethylase–mediated demethylation of suppressive heterochromatin histone H3 lysine 9 tri-methyl (H3K9me3) and lysine 27 tri-methyl (H3K27me3) marks, resulting in the activation of the stress-related NFkB pathway (Calandrelli et al., 2020). Thus, inflammatory stress alters RNA–DNA and heterochromatin marks in the endothelial cells, the changes being partly responsible for endothelial dysfunction.

Innate immune cells such as macrophages are instrumental in secretion of pro-inflammatory cytokines contributing to endothelial dysfunction. But cytokines also act in para- and autocrine manner to cause macrophage polarization into M1—pro-inflammatory and M2—anti-inflammatory populations (Mills et al., 2000). Notably, the differentiation of immune cells is often induced by inflammatory stimuli and infections, marking a very thin line between differentiation cues and stress response, implying that knowledge obtained from developmental studies might be instrumental in understanding the inflammatory stress response. The intriguing process of polarization dependence on the cell cycle and cell cycle-mediated chromatin plasticity was demonstrated recently (Calandrelli et al., 2020). Interleukin-4 (IL-4) is an essential cytokine that promotes the polarization of macrophages into the M2 population, a process instrumental for the suppression of inflammation. Macrophages were induced with IL4 for 24 h (M2), rested for 24 h (M2 primed cells), and then analyzed by a single-cell ATAC-seq method. The state of the chromatin in M2 primed cells differed from that in M0 (naïve macrophages) and M2 cells, suggesting a distinct chromatin organization in all three populations of macrophages. The differentiation of human THP1 monocytes into macrophages is also accompanied by 3D chromatin changes at the level of the TADs (Phanstiel et al., 2017). The stress-related transcription factor complex AP-1 is highly enriched in active hubs in differentiated macrophages when compared to undifferentiated THP1 cells (Phanstiel et al., 2017). Thus, the differentiation of human monocytes into macrophages and the polarization of the macrophages alter 3D chromatin organization at the loop and TAD level.

While the effects of bacterial infections on 3D chromatin organization remain largely unknown, viral infections such as SARS-CoV-2 are shown by using ChIP and Hi-C 3.0, a method that enables the identification of long and short 3D chromatin architecture *in situ*, weakening of euchromatin compartment in the alveolar epithelial-origin A549 cells, and disruption of cohesion loops extrusion (Wang et al., 2021). This is a particularly interesting finding as the long-term effects of SARS-CoV-2 are well described but not explained. Overall, cytokines impact the 3D chromatin organization at the level of TADs and RNA–DNA interactions in both immune and nonimmune cells. In the endothelial (non-immune) cells, TADs remain largely unchanged, but in activated macrophages, changes in TADs have been reported (Figure 1).

## Mechanical Stress Shapes the 3D Chromatin Organization

Cells within tissues undergo mechanical pressure. Additionally, migrating cells such as immune cells are exposed to extreme morphological changes due to increased pressure from the tissue barrier (Martino et al., 2018). Such mechanical stress impacts cytoskeleton organization. The emerging area in the chromatin field is focused on whether mechanical stress alters chromatin architecture. Notably, the cells from multicellular organisms are not the only examples where mechanical pressure affects the chromatin. During mitosis in yeasts, the centromere region of the chromosomes serves as a template to form kinetochores—structures that bind microtubules and enable the separation of chromosomes into daughter cells. The centromeres during mitosis experience significant pulling forces from microtubule movements and display increased histone proteins H2B and H4 turnover in a microtubule-dependent and chromatin remodeling factors–dependent manner (Verdaasdonk et al., 2012). The nucleus senses the extracellular cues through the mechanotransduction pathways (Stephens et al., 2019). Mechanotransduction is the term that collectively describes molecular processes that transform physical cues into biological functions (Martino et al., 2018). The pathways are diverse and include multiple kinases, such as focal adhesion kinases, cytoskeleton tension, and shuttling messengers, that translocate to the nucleus from adhesion sites, such as paxillin (Martino et al., 2018).

The nucleus response to mechanical stress is not limited to cytoskeletal changes but includes the chromatin itself (Stephens et al., 2019). For example, heterochromatin mediates the stiffness of chromatin and is capable of restoring the nuclear shape (Stephens et al., 2019). One of the major proteins that mediates the formation of constitutive heterochromatin and its phase separation, HP1α protein, is essential for chromatin rigidity (Strom et al., 2021). Specifically, auxin-inducible depletion of HP1α from U2OS cells revealed that the crosslinking properties of HP1 are instrumental for nuclear mechanics and shape (Strom et al., 2021). Of interest are the recently reviewed mechanical resistance of various cell types and the role of nuclear stiffness and heterochromatin in response to mechanical stress (Lammerding and Hsia, 2020). Specifically, the unique mechanisms in tissue mechanoadaptation help the epithelial cells to restore H3K9me3-marked heterochromatin, thus preventing mechanical stress–induced DNA damage (Nava et al., 2020). Nuclear envelope protein lamin A mediates the tethering of lamina-associated heterochromatin and is critical for nuclear resistance to mechanical stress. For example, mutations in lamin A lead to cardiac defects due to reduced nuclear stability in cardiomyocytes (Münch and Abdelilah-Seyfried, 2021). Moreover, transient softening of the nucleus by decreasing heterochromatin content via histone deacetylase inhibitor enhanced the cell migration and healing of dense connective tissues in native tissues (Su-Jin et al., 2020). Thus, mechanical stress changes the transcriptional activity in different cell types and alters the physical properties of the heterochromatin (Figure 1), a driver for the nuclear organization (Falk et al., 2019).

## Osmotic Stress

Dehydration of mammalian cells caused by hyperosmotic stress affects their function and chromatin architecture without affecting viability, at least during the first 2 h after exposure to a high sucrose concentration (Olins et al., 2020). One of the most profound defects is the shrinking of the cell volume and alterations in the liquid phase separating nuclear structures such as Ki67 decoration of the nucleolus (Olins et al., 2020). Osmotic stress induces dramatic chromatin changes when human T47D cells are exposed to a final osmolality of 488 mOsm and examined by the Hi-C method and transcriptome analysis. Specifically, the exposure of mammalian cells to osmostress for 60 min resulted in dissociation of the two key chromatin architectural proteins, CTCF and RAD21, from their binding sites, concomitantly in weakened TADs and decreased transcription. These drastic changes, however, are reversed once the cells are placed in isotonic conditions (Amat et al., 2019). Intriguingly, transcription was required for partial recovery, but not for local chromatin changes (Amat et al., 2019). The remarkable changes that are fully reversible have also been reported earlier for chondrocytes (Irianto et al., 2013) and support the notion that quick and dramatic 3D chromatin alterations can be reversed.

## Discussion

We begin to uncover the tip of the iceberg in our understanding of how inflammatory stress impact the spatial organization of the chromatin. Do different cell types react differently to inflammation depending on their function? Based on the initial studies reported here, this might be the case: the altered DNA–RNA interactions but not TADs in the endothelial cells vs. TAD alterations during the polarization of macrophages. This might also be explained by the different stimuli applied. HS also has some documented effects on loops and TAD borders in human embryonic stem cells (Lyu et al., 2018) and invariant TAD boundaries in human somatic cells and *Drosophila* (Ray et al., 2019). Importantly, the chromatin alterations are epigenetically inheritable, at least in *Drosophila* embryos (Seong et al., 2011), suggesting possible mechanisms for the inherited effects of inflammatory stress. Several studies suggest classic stress response mechanisms, such as stress kinases as the mediators of these alterations. Most of the studies, except for a study reporting epigenetic inheritance of the HS-induced chromatin alterations (Seong et al., 2011), use short-term stress, thus the long-term effects remain to be investigated.

The story with inflammatory stress gets complex once we realize that the cells are often exposed to various stress simultaneously: febrile condition usually accompanies inflammatory cytokines; migrating immune cells facing inflammation and mechanical pressure or endothelial cells resisting blood pressure and inflammation. The effect of stress on chromosomal territories has not been investigated till now. These and other questions are under the pressing need to be addressed in this new and exciting field (Stephens et al., 2019; Heo et al., 2020).
